# Nanomanipulation and environmental nanotechnology

**DOI:** 10.3762/bjnano.5.216

**Published:** 2014-11-11

**Authors:** Enrico Gnecco, Andre Schirmeisen, Carlos M Pina, Udo Becker

**Affiliations:** 1 Instituto Madrileño de Estudios Avanzados en Nanociencia (IMDEA Nanociencia), Campus Universitario de Cantoblanco, Calle Faraday 9, 28049 Madrid, Spain; 2 Institute of Applied Physics, Justus-Liebig Universität Giessen, Heinrich-Buff-Ring 16, 35392 Giessen, Germany; 3 Department of Crystallography and Mineralogy, Complutense University of Madrid, 28040 Madrid, Spain; 4 Department of Earth and Environmental Sciences, University of Michigan, Ann Arbor, Michigan 48109-1005, USA

The leitmotif of this Thematic Series is the application of nanotechnology to environmental issues. Since the subject is broad and rapidly evolving it is clearly not possible to completely cover it in the limited space at our disposal. We have rather caught different “flavors” of emerging technologies and presented them as described by the scientists who are actively contributing to their development.

Nanotechnology enters the characterization of processes of environmental interest in a multidisciplinary way. The techniques include atomic force microscopy (AFM), scanning electron microscopy (SEM), transmission electron microscopy (TEM), diffraction methods and advanced chemical analysis. Furthermore, one has often to deal with tiny particles, whose study frequently requires their nanomanipulation. One advantage of scanning probe techniques is that they allow interacting directly with the particles and displacing them in a controlled way on different substrates. In this way, adhesion and friction can be precisely quantified in different environments. Although very few experiments of this kind have been reported so far, the potential of these techniques is enormous.

This Thematic Series is a compilation of papers which provide a wide variety of examples in which nanotechnologies are used to study or potentially solve environmental problems. For example, organic pollutants can be successfully removed from wastewater using the unique catalytic properties of pyrite nanoparticles. Adhesion of marine bacteria can be prevented by new coating materials based on polyethylene oxide. The surface reactivity of minerals in contact with aqueous solutions can be investigated by laser confocal microscopy, as shown on the example of dissolution of the mineral biotite in solutions with acid and basic pH. Recent nanofiltration techniques are reviewed with emphasis on their applicability, costs and up-scaling. A novel gas sensor based on ZnO nanoparticles doped with palladium is presented.

An invaluable support comes from theory, which in combination with experimental techniques can decisively contribute to a better understanding of important nanoscale processes. For example, the photocatalytic degradation of pollutants can be interpreted using density functional theory. On a different scale, AFM measurements in liquid environments can be supported by advanced contact mechanics models including the squeeze-out of wetting fluids.

Adhesion of fluorite nanoparticles on enamel is important in dental applications, as shown by AFM nanomanipulation experiments. The motion of nanoparticles on a surface can be also driven by quartz tuning forks coupled to scanning electron microscopy. As mentioned above, these techniques hold great potential for a better understanding of friction and adhesive forces on the nanoscale.

Last but not least, we would like to mention that this Thematic Series was partially inspired by the “Advanced Materials Science Networking (AMASING)” workshop organized by Prof. Gianaurelio Cuniberti in Da Nang, Vietnam, in March 2013. This was one of the rare occasions to gather together scientists with different background and geographical distribution who are working in the field.

In conclusion, we would like to thank all the researchers who submitted their contributions to the Thematic Series, the reviewers who carefully checked the accuracy of the manuscripts, and the Beilstein Journal of Nanotechnology for providing an open access platform where the scientists involved in such an important subject have found a direct way to disseminate their results at no additional cost.

Enrico Gnecco, Andre Schirmeisen, Carlos M. Pina and Udo Becker

September 2014

